# Electroacupuncture may improve endometrial receptivity in controlled ovarian hyperstimulation mice by activating AVP neurons

**DOI:** 10.3389/fendo.2026.1765673

**Published:** 2026-02-23

**Authors:** Tiantian Ma, Qian Li, Junwei Li, Yi Fang, Yan Zan, Yu Zhuang, Qian Zhu, Liangjun Xia, Youbing Xia

**Affiliations:** 1 Department of Traditional Chinese Medicine, Qinghai University Medical College, Xining, Qinghai, China; 2 School of Acupuncture-Moxibustion and Tuina of Nanjing University of Chinese Medicine, School of Health Preservation and Rehabilitation of Nanjing University of Chinese Medicine, Nanjing, Jiangsu, China; 3 Key Laboratory of Acupuncture and Medicine Research of Minister of Education, Nanjing University of Chinese Medicine, Nanjing, Jiangsu, China; 4 State Key Laboratory of Reproductive Medicine and Offspring Health, Nanjing Medical University, Nanjing, Jiangsu, China

**Keywords:** AVP neurons, clock genes, controlled ovarian hyperstimulation, electroacupuncture, endometrial receptivity

## Abstract

**Background:**

Controlled ovarian hyperstimulation (COH) can lead to reduced endometrial receptivity during the window of implantation (WOI) and disrupted expression of endometrial clock genes. This study aimed to explore the potential mechanisms by which electroacupuncture (EA) improves endometrial receptivity in COH mice, focusing on the regulation of central and peripheral clock genes within the circadian system.

**Methods:**

Experiment 1 included three groups of mice: control (CTR), COH, and EA. Each group was further subdivided into six subgroups according to sampling time. Tissue samples were collected at ZT12-ZT8 on days 4–5 of pregnancy (D4-5). Uterine morphology was examined, and serum estradiol (E2) and progesterone (P4) levels, endometrial receptivity markers, and mRNA expression of circadian clock genes in the hypothalamic-pituitary-ovarian-uterine (HPOU) axis were assessed. In Experiment 2, mice were assigned to five groups: CTR, COH, EA, agonist (AG), and antagonists and EA (AT+EA). All mice were sacrificed at ZT4 on day 5 of pregnancy (D5). We measured arginine vasopressin (AVP) neuropeptide levels in the suprachiasmatic nucleus (SCN); serum E2 and P4 levels; mRNA expression of circadian clock genes in the HPOU axis; and both protein and mRNA levels of endometrial receptivity markers.

**Results:**

EA ameliorates uterine morphological abnormalities in COH mice during the WOI. Furthermore, EA upregulates the mRNA expression of the endometrial receptivity markers, decreases serum P4 levels, and restores the circadian rhythmicity of HPOU axis clock genes. The most significant intergroup differences regarding endometrial receptivity markers were observed at WOI-ZT4. At this time point, both EA and AG interventions increased AVP neuropeptide expression, suppressed serum P4 levels, modulated HPOU axis clock gene expression, and elevated the expression of endometrial receptivity markers at the protein and mRNA levels. Notably, the co-administration of AT with EA attenuated the therapeutic efficacy of EA at this stage.

**Conclusion:**

EA may enhance endometrial receptivity in COH mice at the WOI-ZT4 stage by activating AVP neurons in the SCN and restoring the circadian expression pattern of clock genes along the HPOU axis. These findings provide experimental evidence supporting a circadian mechanism by which EA may enhance endometrial function during assisted reproductive processes.

## 1 Introduction

Infertility has emerged as a global medical challenge, driven by changes in lifestyle and environmental factors. Assisted reproductive technologies, particularly *in vitro* fertilization-embryo transfer, have become the primary therapeutic option for affected individuals (1). COH is a key preparatory procedure for *in vitro* fertilization-embryo transfer. By administering supraphysiological doses of gonadotropins, including follicle-stimulating hormone and luteinizing hormone, COH induces the simultaneous maturation of multiple follicles, thereby facilitating the retrieval of multiple embryos (2). During embryo implantation, there is a specific WOI during which the endometrium exhibits optimal receptivity to the embryo. In mice, the WOI occurs on days 4-5 (D4-5) of pregnancy (3). However, studies have shown that COH can impair endometrial receptivity within the WOI, representing a major cause of implantation failure (4, 5). Currently, there are no definitive therapeutic interventions to effectively address endometrial non-receptivity (6). EA is a therapeutic modality in which fine metal needles are inserted into specific acupoints on the body and electrical stimulation is applied to modulate meridians, regulate the flow of Qi and Blood, harmonize Yin and Yang (7). Both clinical and animal studies have demonstrated that EA can modulate the hypothalamic-pituitary-ovarian axis to promote ovulation, enhance endometrial receptivity, and improve embryo quality, thereby increasing clinical pregnancy rates (8–13).

In recent years, the interplay between circadian rhythms and reproductive function has gained increasing attention (14, 15). The HPOU axis orchestrates female reproductive processes, while disruptions in circadian rhythms may impair this axis, and result in ovarian and uterine dysfunction (16, 17). Mammalian circadian rhythms are governed by the coordinated activity of central and peripheral clocks (18). The SCN functions as the central pacemaker of circadian rhythms and consists predominantly of two types of neuropeptide-rich neurons: AVP neurons and vasoactive intestinal peptide neurons. AVP neurons are located in the shell region of the SCN, which exhibits the most robust rhythmic expression of clock genes, and they are critical for the generation and pacing of circadian rhythms within the SCN network (19). Evidence suggests that acupuncture can modulate circadian rhythm disturbances by influencing both the amplitude and phase of clock gene and protein expression within the SCN and peripheral tissues (20, 21). However, it remains unclear whether the mechanisms by which acupuncture improves endometrial receptivity are related to circadian regulation. Therefore, in this study, a COH mouse model was established to investigate the effects of EA on AVP neurons and on peripheral clock genes along the HPOU axis, and to elucidate the potential mechanisms through which it enhances endometrial receptivity during the WOI in COH mice.

## 2 Materials and methods

### 2.1 Animals and grouping

C57BL/6 wild-type female mice (8–10 weeks old, weighing 18–20 g) were used to establish COH models. C57BL/6 wild-type male mice (12–16 weeks old, weighing 25–30 g) were castrated and employed for mating with the females. All mice were obtained from Jiangsu Huachuang Xinnuo Pharmaceutical Technology Co., Ltd. and housed in a specific pathogen-free facility under controlled environmental conditions: temperature maintained at 22 ± 2 °C, relative humidity at 40-60%, and a 12: 12 h light-dark cycle (lights on at 08:00 and off at 20: 00). Mice had free access to food and water and underwent a 2-week acclimation period before the experiment. All experimental procedures were reviewed and approved by the Animal Ethics Committee of Nanjing University of Chinese Medicine (Approval No. 202309A067). Female mice were monitored daily for estrous cycles via vaginal cytology. Only mice exhibiting two consecutive regular estrous cycles were included in subsequent experiments. In Experiment 1, female mice were assigned to three groups: CTR, COH, and EA. Each group was further divided into six subgroups according to sampling time: ZT12 (20: 00), ZT16 (0: 00), ZT20 (04: 00), ZT0 (08: 00), ZT4 (12: 00), ZT8 (16: 00). In Experiment 2, animals were allocated into five groups: CTR, COH, EA, AG, and AT+EA.

### 2.2 COH modeling and experimental procedures

The COH protocol was conducted following established experimental methods described in previous studies (22, 23). Female C57BL/6 mice in the COH, EA, AG, and AT+EA groups received intraperitoneal injections of PMSG (5 U/mouse, Ningbo Second Hormone Factory, Veterinary Drug Registration No. 110254564) at 17: 00 during the diestrus. After 48 hours, the mice were administered an intraperitoneal injection of HCG (5 U/mouse, Ningbo Second Hormone Factory, Veterinary Drug Registration No. 110251282). Mice in the CTR group received an equal volume of 0.9% saline at the corresponding time points. Subsequently, female mice were housed overnight with vasectomized males in individual cages for mating. To eliminate the influence of embryos on the uterine endometrium after conception, males were vasectomized prior to pairing, and successful copulation induced a pseudopregnant state in the females. Mating success was confirmed by the presence of a vaginal plug at 08:00 the following morning, which was designated as gestation day 1 (D1). Only female mice that successfully mated were included in the experiment.

**Figure 1:**
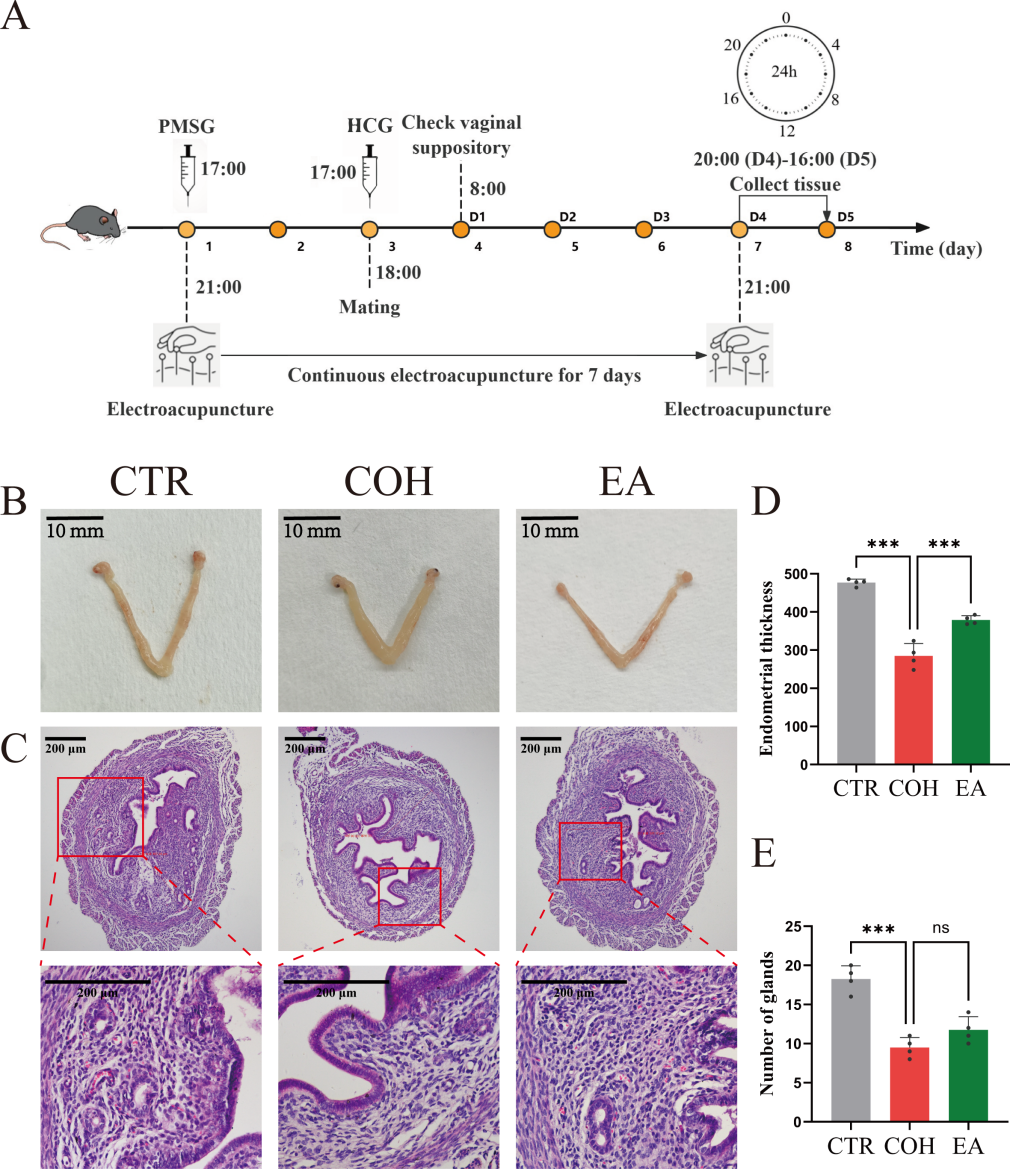
EA ameliorates uterine morphological abnormalities in COH mice during the WOI. **(A)** Experimental workflow diagram for Experiment 1. **(B)** Uterine morphology of mice, scale bar: 10 mm. **(C)** Representative HE-stained uterine sections of mice, scale bar: 200 μm. **(D)** Endometrial thickness in mouse uteruses (n = 4). **(E)** Number of uterine glands in mice (n = 4). ***P < 0.001, **P < 0.01, *P < 0.05, ns, *P* > 0.05.

Female rats in the AG group received intraperitoneal injections of the AVP Receptor 1A (AVPR1A) agonist: (Phe2, Orn8)-Oxytocin (1.0 mg/kg, MCE, HY-P4678), starting on the day of PMSG administration and given once daily at 17:30 for 7 consecutive days (24, 25). Female rats in the AT+EA group received intraperitoneal injections of the AVPR1A antagonist: SR49059 (1.0 mg/kg, MCE, HY-18345) at the same time as (Phe2, Orn8)-Oxytocin (26). For the EA and AT+EA groups, mice were anesthetized before receiving EA at bilateral SP6 and CV4 acupoints using disposable acupuncture needles (0.18 × 13 mm; Beijing Zhongyan Taihe Medical Devices Co., Ltd). Needles were inserted perpendicularly to a depth of approximately 5 mm. EA was delivered via an electronic acupuncture device (Suzhou Medical Supplies Factory Co., Ltd, SDZ-II) with a sparse-dense waveform at a 2 Hz/15 Hz frequency and intensity adjusted to induce mild twitching of the hind limbs. During each electroacupuncture session, electrical stimulation was applied between CV4 and one-sided SP6, while the contralateral SP6 was needled without electrical stimulation. Start electroacupuncture at 21:00 on the day of PMSG injection, once daily for 15 minutes each time, and continue for 7 consecutive days. Mice in all other groups were anesthetized at the same time points without further intervention (**Figure 1A**). As mice are nocturnal animals, 21:00 falls within their active period, making electroacupuncture at this time more consistent with their natural living habits.

### 2.3 Tissue collection and sample processing

Experimental mice were dissected at designated time points according to their subgroup assignments between D4-5. Sampling was performed at six ZT points: ZT12, ZT16, ZT20, ZT0, ZT4, and ZT8. Following anesthesia, blood samples were collected from the retinal vein, and mice were subsequently euthanized via cervical dislocation. Serum was separated by centrifugation and stored for enzyme-linked immunosorbent assay (ELISA) to determine E2 and P4 levels. Tissue samples, including the hypothalamus, pituitary gland, ovaries, and unilateral uterus, were rapidly harvested and stored at −80 °C for subsequent molecular analyses. The contralateral uterus was fixed in 4% paraformaldehyde and stored at 4 °C for HE staining and immunohistochemical analysis. In Experiment 2, mice were sacrificed at ZT4 (12: 00) on D5. After anesthesia, animals underwent cardiac perfusion, and the whole brain was fixed in 4% paraformaldehyde. The brains were then processed for sectioning and subsequent immunofluorescence staining.

### 2.4 HE staining

Fresh uterine tissue was immediately fixed in 4% paraformaldehyde for more than 24 hours. Embed the tissue in paraffin blocks, section it into 5-micrometer slices, mount the sections onto slides, and air-dry them for later use. The tissue sections underwent a series of histological procedures, including dewaxing, staining, dehydration, and sealed. Observe the sections under an optical microscope (Olympus Corporation, BX53) and collect images.

### 2.5 Enzyme-linked immunosorbent assay

Serum concentrations of E2 and P4 were determined using the ELISA method. The following commercial kits were used: Mouse Estradiol ELISA Kit (Afantibody, AF02457-A) and Mouse Progesterone ELISA Kit (Afantibody, AF02568-A). All procedures were performed strictly in accordance with the manufacturer’s instructions. After completing the assays, absorbance values were measured using a microplate reader, and hormone concentrations were calculated based on the corresponding standard curves.

### 2.6 Immunohistochemistry staining

Paraffin sections are deparaffinized in xylene, then placed in gradient concentrations of ethanol for antigen retrieval. Endogenous peroxidase activity was quenched using 3% hydrogen peroxide (H_2_O_2_) in deionized water for 10 minutes. Antigen retrieval was performed in citrate buffer using heat-induced epitope retrieval for 23 minutes, followed by blocking with 5% bovine serum albumin at room temperature for 30 minutes to reduce nonspecific binding. Primary antibodies were applied and incubated overnight at 4 °C: Homeobox A10 (HOXA10, 1: 100 dilution, Abclonal, A8550) and Secreted Phosphoprotein 1 (SPP1, 1: 100 dilution, Abmart, T55333). The following day, sections were incubated with a biotin-labeled goat anti-rabbit IgG secondary antibody (1: 200 dilution, Boster, BA1003) at room temperature for 30 minutes. Subsequently, an SABC signal amplification reagent was added and incubated for 30 minutes at room temperature. Visualization was achieved using a DAB chromogen reaction for 10 minutes, followed by hematoxylin counterstaining for 3 minutes. Finally, the sections are dehydrated, cleared, and sealed. Stained slides were observed under a light microscope, and digital images were captured. The staining intensity and positive area were quantitatively analyzed using ImageJ software to evaluate protein expression levels.

### 2.7 Immunofluorescence staining

Paraffin sections were first dewaxed in xylene and rehydrated through a series of graded ethanol solutions for antigen retrieval. The sections were then treated with citrate buffer for antigen retrieval. After antigen retrieval, peroxidase inhibitor solution was applied to block endogenous peroxidase activity, followed by incubation with normal goat serum to block non-specific binding sites. The sections were incubated overnight at 4 °C with the primary antibody AVP (1: 2000 dilution, Immunostar, 20069). Following primary antibody incubation, a Polymer-HRP secondary antibody was added and incubated for 30 minutes at room temperature in the dark. Subsequently, add TYR-520 fluorescent dye dropwise, and incubate at room temperature for 5–10 minutes. Finally, a fluorescence quenching mounting medium containing DAPI was added, and the slides were allowed to stand for approximately 5 minutes. The stained sections were examined under a fluorescence microscope, and digital images were captured. Staining intensity and positive area were analyzed quantitatively using ImageJ software to assess the expression levels of AVP. All reagents used for this procedure were obtained from the Single Label Signal Amplification Fluorescent Staining Kit (Afantibody, AFIHC022).

### 2.8 Western blotting

Tissue samples were minced and lysed in RIPA buffer for 10 minutes on ice. Lysates were then centrifuged at 12,000 rpm for 15 minutes at 4 °C, and the supernatant was collected. Protein concentrations were quantified using a microplate reader. Equal amounts of protein were mixed with SDS-PAGE loading buffer and denatured by boiling. Proteins were separated by SDS-PAGE electrophoresis and subsequently transferred onto polyvinylidene difluoride membranes. The membranes were blocked with 5% bovine serum albumin at room temperature for 1 hour to prevent nonspecific binding. Membranes were incubated overnight at 4 °C with primary antibodies against HOXA10 (1: 1000 dilution, Abclonal, A8550), SPP1 (1: 1000 dilution, Abmart, T55333), and β-actin (1: 5000, Proteintech, 20536-1-AP). After washing, membranes were incubated with appropriate HRP-conjugated secondary antibodies at room temperature for 1 hour. Protein bands were visualized using a chemiluminescent imaging system (VILBER BIO IMAGING, FUSION-FX6.MINI), and band intensities were quantified by ImageJ software to calculate the relative expression levels of target proteins normalized to β-actin.

### 2.9 Real-time quantitative PCR

Tissue samples were processed with Trizol to extract total RNA. Chloroform was then used to remove proteins and lipids, followed by isopropanol precipitation of RNA. The RNA pellets were washed with ethanol, and RNA concentration and purity were assessed using a spectrophotometer. cDNA was synthesized using a reverse transcription kit (Yeasen, 11141ES60). PCR amplification was carried out with a pre-mixed solution kit (Yeasen, 11202ES08) on a real-time quantitative PCR system (Thermo Fisher Scientific, ViiA 7). Gene-specific primers are listed in **Table 1**. Relative gene expression was calculated using the 2^−ΔΔCT^ method, with CT values normalized to the reference gene *Gapdh*.

**Table 1:** Primer sequences used for quantitative reverse transcription polymerase chain reaction.

Gene symbol	Forward primer	Reverse primer	Product length (bp)
*Gapdh*	AACTTTGGCATTGTGGAAGGG	GACACATTGGGGGTAGGAACA	224
*Hoxa10*	CCTAGAGATCAGCCGTAGCG	GAGCTCCCGGATTCGGTTTT	112
*Spp1*	AGGACAACAACGGAAAGGGC	CATCCGACTGATCGGCACTC	114
*Maoa*	GCCCTGTGGTTCTTGTGGTA	GGCCAGAGCCACCTACAAAT	103
*Per1*	TCCCCTATTCGCTTCTGTGC	TTTATGGCGACCCAACACGA	114
*Cry1*	TGCCCTGTGGGTTTTGGTAG	TGCCTTCTGGTGCATTCCAA	121
*Cry2*	CACTGGTTCCGCAAAGGACTA	CCACGGGTCGAGGATGTAGA	102
*Fbxl3*	GGACTTATCTCGACTGCCCG	TCGACTGGGGTGTCGTCTAT	128
*Csnk1e*	ACCTAGAGAGCTTGGGCTAT	CTAATCCGCTCGTACTTCTGAC	103

### 2.10 Statistical analysis

Statistical analysis was conducted using GraphPad Prism 10.4.1 software. Data are expressed as mean ± standard error. The Shapiro-Wilk test was employed to assess the normality of the data. For comparisons between groups at a single time point, One-way analysis of variance was used. If the data met the assumption of homogeneous variances, Tukey’s multiple comparison test was applied to determine pairwise differences. If variances were found to be heterogeneous, Brown-Forsythe and Welch’s analysis of variance tests were used to reanalyze the data. For comparisons across multiple time points, two-way analysis of variance was conducted, followed by Dunnett’s multiple comparison test to assess specific group differences. For data that did not follow a normal distribution, nonparametric tests were applied. A *P* < 0.05 was considered statistically significant.

## 3 Result

### 3.1 EA ameliorates uterine morphological abnormalities in COH mice during the WOI

To evaluate the effect of EA on uterine morphology during the WOI in COH mice, we first examined the gross appearance of the uterus and then performed HE staining to assess histological changes, including quantitative analysis of endometrial thickness and gland number. Compared with the CTR group, uteri from COH mice appeared markedly thickened and edematous with a pale coloration. The endometrial architecture was disorganized, the endometrium was thinner, and both the number of glands and the glandular lumen diameter were reduced. In contrast, relative to the COH group, uteri from EA-treated mice were slimmer with attenuated edema, and showed significantly increased endometrial thickness and gland numbers (**Figures 1B–E**). These findings indicate that EA ameliorates COH-induced uterine morphological abnormalities.

### 3.2 EA mitigates the elevated P4 levels and restores endometrial receptivity in COH mice during the WOI

**Figure 2:**
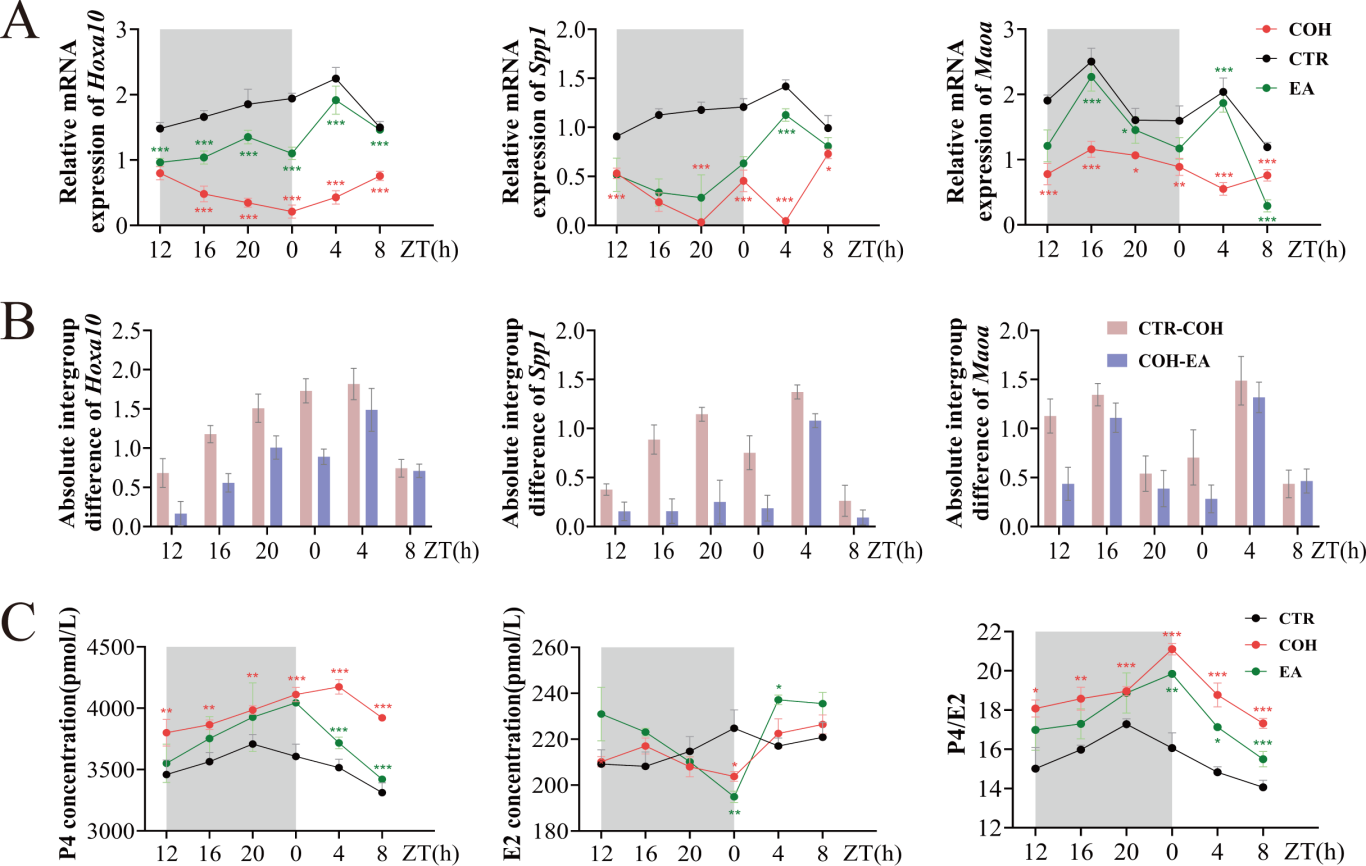
EA reverses the surge in P4 levels and the impairment of endometrial receptivity during the WOI in COH mice. **(A)***Hoxa10*, *Spp1*, and *Maoa* mRNA levels in mice during the WOI from ZT12 to ZT8 (n = 4). **(B)** Absolute intergroup differences in *Hoxa10*, *Spp1*, and *Maoa* mRNA levels in mice during the WOI from ZT12 to ZT8. **(C)** Serum P4, E2, and P4/E2 levels in mice during the WOI from ZT12 to ZT8 (n = 4).* indicates significant difference between COH and CTR groups; * indicates significant difference between EA and COH groups. ***P < 0.001, **P < 0.01, *P < 0.05, ns, *P* > 0.05.

Endometrial thickness and gland number are key histological parameters for evaluating endometrial receptivity. The histological findings described above suggested that EA may improve endometrial receptivity. To further clarify the effect of EA on endometrial receptivity during the WOI in COH mice, we assessed uterine mRNA levels of the receptivity markers *Hoxa10*, *Spp1*, and Monoamine Oxidase A (*Maoa*) at six time points from ZT12 to ZT8. Compared with the CTR group, COH mice showed reduced *Hoxa10*, *Spp1*, and *Maoa* mRNA levels across ZT12-ZT8. In contrast, relative to the COH group, EA-treated mice exhibited increased *Hoxa10*, *Spp1*, and *Maoa* mRNA levels at the same time points (**Figure 2A**). These data indicate that COH impairs endometrial receptivity, whereas EA can partially restore it.

To determine the time point at which EA exerts the most pronounced effect on WOI endometrial receptivity in COH mice, we compared, for each of the six Zeitgeber times from ZT12 to ZT8, the absolute differences in *Hoxa10*, *Spp1*, and *Maoa* mRNA levels between the CTR and COH groups, as well as between the COH and EA groups. Both sets of absolute differences reached their maximum at ZT4 (**Figure 2B**). This finding suggests that endometrial receptivity during the WOI is most severely impaired by COH at ZT4, and that the beneficial effect of EA on COH-induced impairment is also greatest at this time point. Accordingly, ZT4 was selected as the observation time point for subsequent experiments.

P4-induced secretory transformation is a central mechanism underlying the acquisition of endometrial receptivity (27). The P4/E2 ratio during the WOI reflects the degree of synchronization between the uterine microenvironment and embryonic development and provides a more accurate predictor of pregnancy outcome (28, 29). To evaluate the influence of EA on P4 and E2 levels during the WOI in COH mice, we measured serum P4 and E2 concentrations at ZT12-ZT8 and calculated the P4/E2 ratio. Compared with the CTR group, COH mice showed significantly increased serum P4 levels and P4/E2 ratios across ZT12-ZT8. EA treatment markedly reduced serum P4 levels and P4/E2 ratios relative to the COH group, suggesting that EA may help normalize sex steroid hormone levels in COH mice. In contrast, no significant differences in serum E2 levels were observed among the three groups (**Figure 2C**).

### 3.3 EA restores circadian rhythm disruption in COH mice with disrupted WOI-HPOU axis clock genes

Circadian variation in core clock genes plays a critical role in regulating endometrial receptivity during the WOI (30). To investigate the effect of EA on the circadian expression of clock genes along the WOI-HPOU axis in COH mice, we measured mRNA levels of the clock genes Period Circadian Regulator 1 (*Per1*), Cryptochromes 1 and 2 (*Cry1, Cry2*), and their key regulatory factors F-box and Leucine-Rich Repeat Protein 3 (*Fbxl3*) and Casein Kinase 1ϵ (*Csnk1e*) in the hypothalamus, pituitary, ovary, and uterus at six Zeitgeber time points from ZT12 to ZT8.

**Figure 3:**
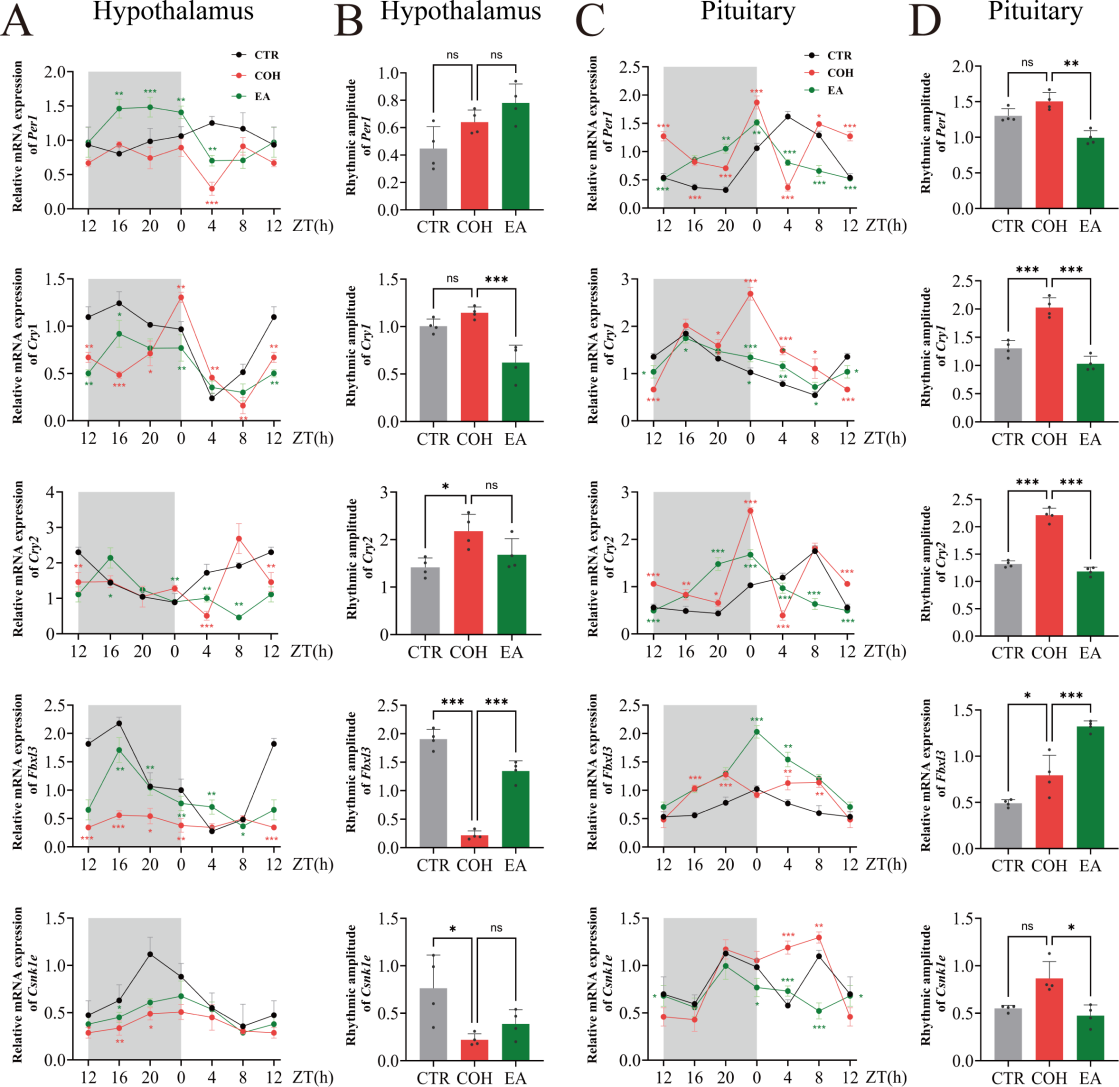
EA restores the circadian rhythmicity of clock gene expression in the hypothalamic and pituitary gland during the WOI in COH mice. **(A, B)** Hypothalamic *Per1*, *Cry1*, *Cry2*, *Fbxl3* and *Csnk1e* mRNA levels and circadian amplitudes in mice during the WOI from ZT12 to ZT8 (n = 4). **(C, D)** Pituitary gland *Per1*, *Cry1*, *Cry2*, *Fbxl3* and *Csnk1e* mRNA levels and circadian amplitudes in mice during the WOI from ZT12 to ZT8 (n = 4). * indicates significant difference between COH and CTR groups; * indicates significant difference between EA and COH groups. ***P < 0.001, **P < 0.01, *P < 0.05, ns, *P* > 0.05.

The results showed that in mice from the CTR group, hypothalamic and pituitary mRNA expression of *Per1*, *Cry1*, *Cry2*, *Fbxl3*, and *Csnk1e* exhibited regular circadian rhythmicity. In contrast, in the COH group, the circadian rhythmicity of these genes in the hypothalamus and pituitary was disrupted; notably, the rhythmic expression of hypothalamic *Fbxl3* and *Csnk1e* mRNA was lost and the amplitude was markedly reduced. In the EA group, hypothalamic and pituitary *Per1*, *Cry1*, *Cry2*, *Fbxl3*, and *Csnk1e* mRNA expression regained clear circadian rhythmicity, and the phases of the peak and trough of *Cry1* mRNA rhythms were consistent with those in the CTR group (**Figures 3A–D**). This indicates that circadian rhythmicity of hypothalamus and pituitary-related clock genes is disrupted in COH mice during the WOI stage, while EA can improve and partially restore these rhythmic changes.

**Figure 4:**
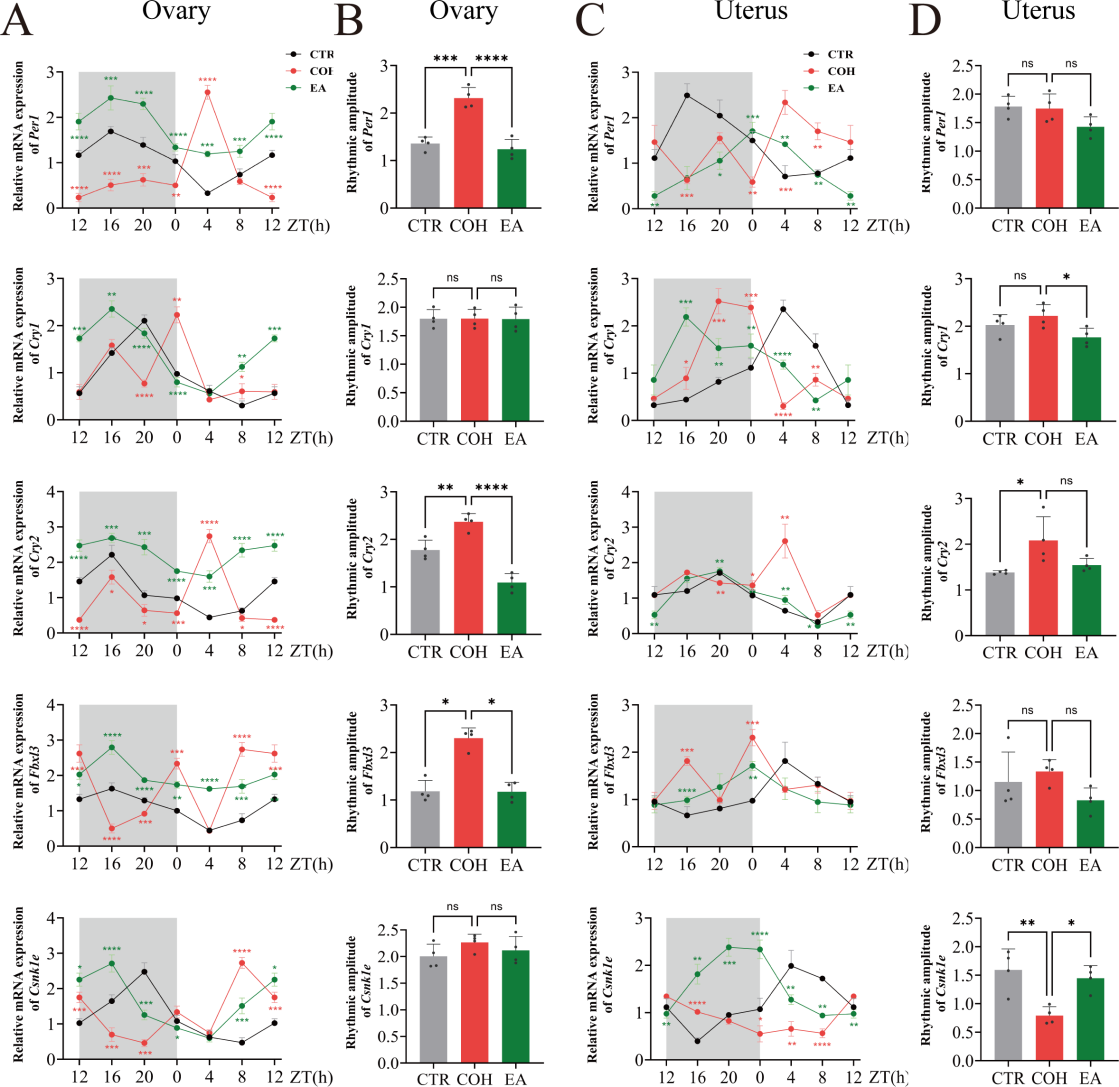
EA restores the circadian rhythmicity of clock gene expression in the ovary and uterus during the WOI in COH mice. **(A, B)** Ovarian *Per1*, *Cry1*, *Cry2*, *Fbxl3* and *Csnk1e* mRNA levels and circadian amplitudes in mice during the WOI from ZT12 to ZT8 (n = 4). **(C, D)** Uterine *Per1*, *Cry1*, *Cry2*, *Fbxl3* and *Csnk1e* mRNA levels and circadian amplitudes in mice during the WOI from ZT12 to ZT8. (n = 4). * indicates significant difference between COH and CTR groups; * indicates significant difference between EA and COH groups. ***P < 0.001, **P < 0.01, *P < 0.05, ns: *P* > 0.05.

In line with the changes observed in the hypothalamus and pituitary, ovarian and uterine *Per1*, *Cry1*, *Cry2*, *Fbxl3*, and *Csnk1e* mRNA expression in the CTR group displayed regular circadian rhythmicity. In COH mice, however, the circadian rhythmicity of these genes in the ovary and uterus was disrupted, whereas EA treatment restored their rhythmic expression. In the ovaries of CTR and EA mice, *Per1*, *Cry1*, *Cry2*, *Fbxl3*, and *Csnk1e* mRNA levels showed similar peak and trough phases, with peak expression occurring between ZT16 and ZT20 and trough expression between ZT4 and ZT8 (**Figures 4A–D**). Taken together, these findings indicate that COH disrupts the circadian rhythmicity of *Per1*, *Cry1*, *Cry2*, *Fbxl3*, and *Csnk1e* expression along the HPOU axis during the WOI, whereas EA restores their circadian rhythmic patterns.

### 3.4 EA enhances AVP neuropeptide expression in the SCN of COH mice at WOI-ZT4

**Figure 5:**
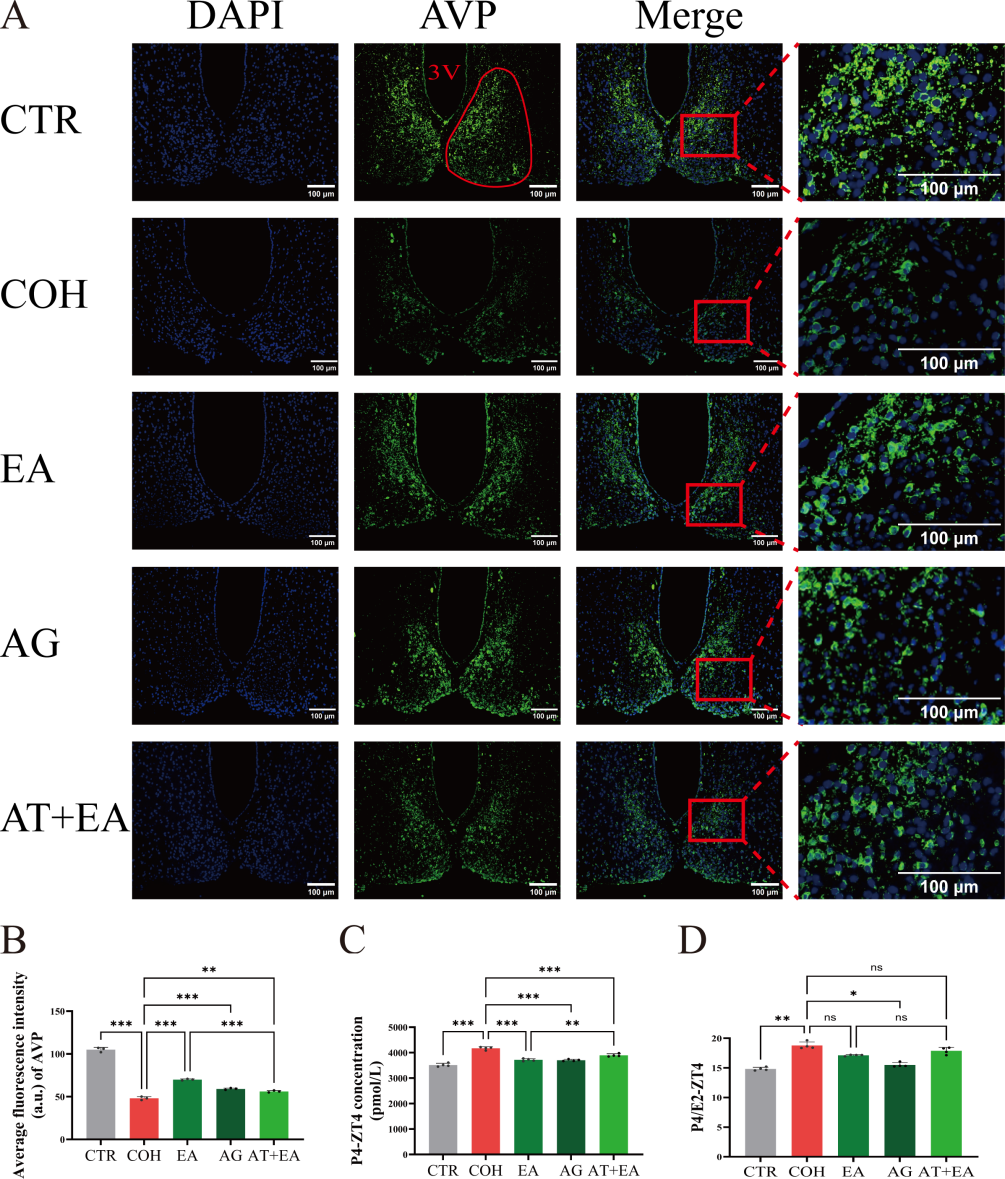
EA enhances AVP neuropeptide expression in the SCN and reduces serum hormone levels at WOI-ZT4 in COH mice. **(A, B)** Representative immunofluorescence images and mean staining intensity of AVP neuropeptides in the SCN at WOI-ZT4 in mice (n = 3). Scale bar: 100 μm. **(C, D)** Serum P4 levels and P4/E2 ratios at WOI-ZT4 in mice (n = 4). 3V: Third ventricle. The SCN is indicated by the red circle in the figure. AVP fluorescence staining is predominantly distributed in the marginal region of the SCN. DAPI: Cell nuclei; Merge: Merged channels. ***P < 0.001, **P < 0.01, *P < 0.05, ns, *P* > 0.05.

The central circadian clock in the SCN is a network composed of neurons and glial cells, among which AVP-expressing neurons act as the principal pacemakers of the SCN clock network (31). To examine the effect of EA on the central clock at WOI-ZT4 in COH mice, we performed immunofluorescence staining for AVP neuropeptide in the SCN of the hypothalamus to assess AVP neuronal activity. The results showed that, compared with the CTR group, AVP neuropeptide expression at WOI-ZT4 was markedly reduced in the COH group. Relative to the COH group, AVP neuropeptide expression intensity at WOI-ZT4 was increased in both the EA and AG groups. In contrast, AVP neuropeptide expression at WOI-ZT4 was reduced in the AT+EA group compared with the EA group (**Figures 5A, B**).

In parallel, we measured serum P4 levels and the P4/E2 ratio at WOI-ZT4 to evaluate the hormonal status of COH mice. Compared with the CTR group, COH mice exhibited elevated serum P4 concentrations and P4/E2 ratios at WOI-ZT4. Relative to the COH group, both the EA and AG groups showed reduced serum P4 levels and P4/E2 ratios. Notably, serum P4 levels and the P4/E2 ratio were higher in the AT+EA group than in the EA group (**Figures 5C, D**). Collectively, these findings demonstrate that COH mice exhibit reduced AVP neuropeptide expression and hormone levels in the hypothalamic SCN at WOI-ZT4. EA and AVPR1A agonist treatment can improve these alterations, while AVPR1A antagonist combined with EA treatment attenuates the modulatory effect of EA.

### 3.5 EA improves abnormal expression of HPOU-axis clock genes at WOI-ZT4 in COH mice

Because AVP neurons in the central clock of the SCN can regulate peripheral clocks, we next examined mRNA expression of the clock genes *Per1*, *Cry1*, and *Cry2* in the hypothalamus, pituitary, ovary, and uterus at WOI-ZT4. Compared with the CTR group, COH mice showed decreased *Per1* and *Cry2* mRNA expression in the hypothalamus and pituitary, and reduced *Cry1* mRNA expression in the ovary and uterus at WOI-ZT4, whereas the other clock genes were upregulated. These findings indicate that COH disrupts the normal expression pattern of key clock genes along the HPOU axis at this time point.

**Figure 6:**
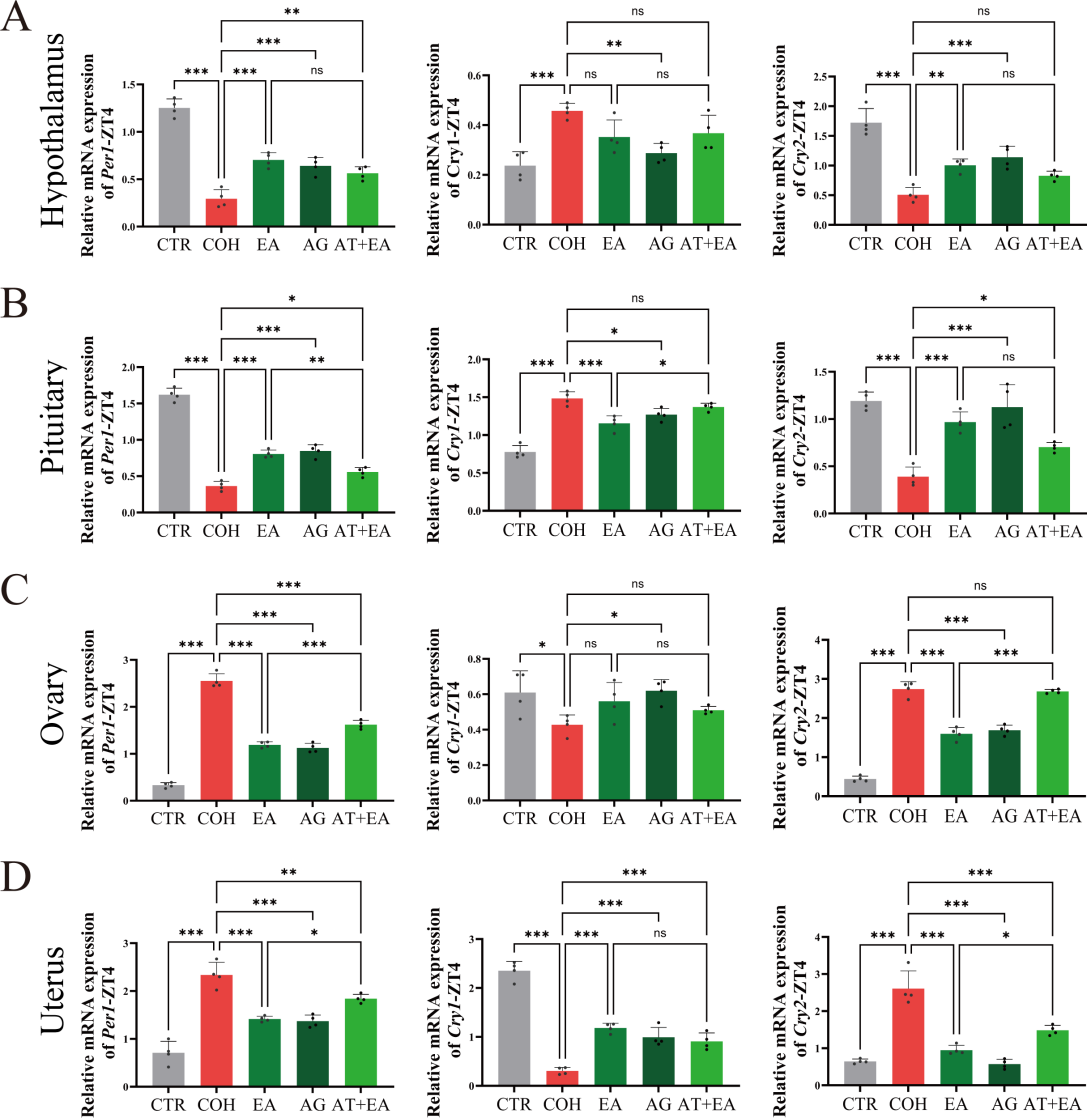
EA can improve abnormal expression of HPOU axis at WOI-ZT4 in COH mice. **(A)** Hypothalamic *Per1*, *Cry1*, and *Cry2* mRNA levels in mice at WOI-ZT4 (n = 4). **(B)** Pituitary gland *Per1*, *Cry1*, and *Cry2* mRNA levels in mice at WOI-ZT4 (n = 4). **(C)** Ovarian *Per1*, *Cry1*, and *Cry2* mRNA levels in mice at WOI-ZT4 (n = 4). **(D)** Uterine *Per1*, *Cry1*, and *Cry2* mRNA levels in mice at WOI-ZT4 (n = 4). ***P < 0.001, **P < 0.01, *P < 0.05, ns, *P* > 0.05.

By contrast, in the EA and AG groups, *Per1* and *Cry2* mRNA levels in the hypothalamus and pituitary, as well as *Cry1* mRNA levels in the ovary and uterus, were increased relative to the COH group, while the other clock genes were altered in the opposite direction. Compared with the EA group, the AT+EA group exhibited reduced *Per1* and *Cry2* mRNA expression in the hypothalamus and pituitary, and decreased *Cry1* mRNA levels in the ovary and uterus at WOI-ZT4, accompanied by opposite changes in the remaining clock genes (**Figures 6A–D**).

Taken together, these results show that in COH mice at WOI-ZT4, *Per1* and *Cry2* mRNA levels are reduced in the hypothalamus and pituitary, and *Cry1* mRNA levels are reduced in the ovary and uterus, whereas hypothalamic and pituitary *Cry1*, and ovarian and uterine *Per1* and *Cry2* mRNA levels are increased. EA and AVPR1A agonist treatment can improve the abnormal expression of HPOU axis clock genes in COH mice during the WOI-ZT4 period, whereas co−administration of an AVPR1A antagonist attenuates this regulatory effect of EA on gene expression.

### 3.6 EA enhances endometrial receptivity at WOI-ZT4 in COH mice

**Figure 7:**
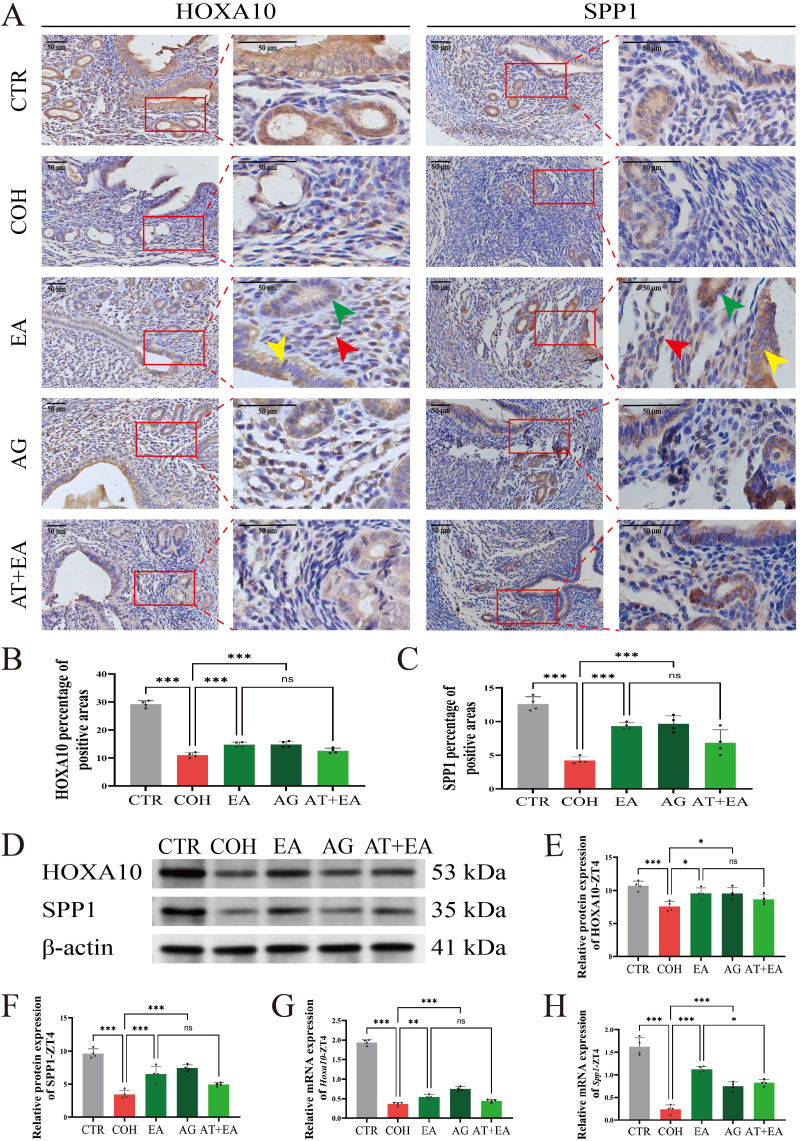
EA improves endometrial receptivity in COH mice at WOI-ZT4. **(A)** Representative immunohistochemical images of uterine HOXA10 and SPP1 expression in mice at WOI-ZT4 (n=4), scale bar: 50 μm. The green arrows indicate endometrial glandular epithelial cells, the red arrows indicate endometrial stromal cells, and the yellow arrows indicate luminal epithelial cells of the endometrium. **(B, C)** Percentage of HOXA10 and SPP1 positive areas in uterine immunohistochemical staining in mice at WOI-ZT4 (n=4). **(D)** Representative western blotting images of uterine HOXA10 and SPP1 protein expression in mice at WOI-ZT4 (n=4). **(E, F)** Grayscale values of uterine HOXA10 and SPP1 protein expression in mice at WOI-ZT4. **(G, H)** Relative uterine *Hoxa10* and *Spp1* mRNA expression levels in mice at WOI-ZT4 (n=4). ***P < 0.001, **P < 0.01, *P < 0.05, ns, *P* > 0.05.

HOXA10 and SPP1 exhibit dynamic changes in expression in endometrial epithelial and stromal cells (32, 33). To examine the expression of HOXA10 and SPP1 in the endometrium of mice at WOI-ZT4, we performed immunohistochemical staining. The results showed that at WOI-ZT4, HOXA10 and SPP1 were positively expressed in endometrial glandular epithelial cells, stromal cells, and luminal epithelial cells (**Figure 7A**).

Quantitative immunohistochemical analysis revealed that compared with the CTR group, HOXA10 and SPP1 protein levels at WOI-ZT4 were reduced in the COH group. Compared with the COH group, HOXA10 and SPP1 protein expression at WOI-ZT4 was increased in the EA and AG groups. Compared with the EA group, the AT+EA group showed decreased expression of the endometrial receptivity markers HOXA10 and SPP1 at WOI-ZT4 (**Figures 7B, C**).

Western blotting and RT-qPCR analyses confirmed that the expression patterns of HOXA10 and SPP1 protein and mRNA in each group were consistent with the immunohistochemical findings (**Figures 7D–H**). These results indicate that COH mice exhibit impaired endometrial receptivity at WOI-ZT4, while both EA and AVPR1A agonist treatment improve endometrial receptivity-related markers. Co−administration of the AVPR1A antagonist with EA attenuates this ameliorative effect.

## 4 Discussion

In this study, a COH mouse model was successfully established by intraperitoneal injection of PMSG and HCG. The experimental results demonstrated that EA intervention significantly ameliorated endometrial injury during the WOI in COH mice and effectively corrected circadian disruption of clock genes along the HPOU axis. Mechanistic investigations revealed that EA enhanced endometrial receptivity in COH mice by potentially activating AVP neurons in the central circadian pacemaker, the SCN, during the WOI-ZT4 time window. This activation modulated the expression of clock genes in peripheral tissues along the HPOU axis and restored physiological serum levels of P4 and the P4/E2 ratio, thereby improving uterine endometrial receptivity.

Although COH can promote the development of multiple follicles, supraphysiological levels of E2 and P4 disrupt the synchrony between endometrial maturation and embryonic development. Histologically, abnormal development of endometrial glands and pinopodes has been observed, while at the molecular level, impaired angiogenesis and increased vascular permeability mediated by human leukocyte antigen F and vascular endothelial growth factor have been reported, ultimately leading to reduced endometrial receptivity (34–36). Current clinical strategies to improve receptivity include endometrial scratching (37, 38), intra-vaginal administration of granulocyte colony-stimulating factor (39, 40), and use of DNA methylation inhibitors (41, 42). However, due to the lack of large-scale randomized controlled trials in humans, the clinical efficacy of these interventions remains uncertain.

Acupuncture, a fundamental component of traditional Chinese medicine, has gained worldwide recognition for its safety and efficacy in treating various disorders. Under the guidance of traditional Chinese medicine meridian theory, EA offers higher precision and controllability than manual acupuncture (43). Previous studies have shown that acupuncture at SP6 and CV4 can enhance endometrial receptivity by improving uterine angiogenesis, promoting migration and paracrine effects of bone marrow-derived stem cells toward the injured uterus, and modulating inflammatory cytokines and the endometrial immune microenvironment (9, 44–46). These findings are highly consistent with the traditional Chinese medicine theory of “regulating the Chong and Ren vessels” SP6 harmonizes the Qi and Blood of the liver, spleen, and kidney meridians, whereas CV4 is a key acupoint on the Ren vessel; their combined stimulation can effectively correct dysfunction of the “kidney-Tian Gui-Chong-Ren-uterus” axis. On this basis, we selected SP6 and CV4 to investigate the circadian rhythm–related mechanisms by which EA improves endometrial receptivity in COH mice.

In the present study, EA markedly alleviated uterine edema in COH mice, which may be related to the regulation of capillary permeability (8, 47, 48). Histological analysis further revealed that EA increased endometrial thickness and gland number—two widely accepted histomorphological parameters for evaluating endometrial receptivity (49, 50). These observations suggested that EA might enhance endometrial receptivity during the WOI in COH mice, prompting us to examine the expression of the receptivity markers *Hoxa10*, *Spp1*, and *Maoa* by RT-qPCR. HOXA10, a transcription factor, plays a central role in regulating endometrial proliferation, differentiation, and decidualization (51–53). SPP1 is a secreted extracellular matrix protein, and MAOA is a mitochondrial outer membrane enzyme; both have been repeatedly identified as WOI-specific receptivity genes in multiple transcriptomic studies (54–56). In our study, EA reversed the COH-induced downregulation of *Hoxa10*, *Spp1*, and *Maoa* mRNA levels, providing direct molecular evidence that EA improves endometrial receptivity.

P4 plays a crucial role in female reproductive physiology by regulating key processes including oocyte maturation, ovulation, endometrial decidualization, and parturition, thereby promoting conception and supporting pregnancy (57). However, abnormally elevated P4 levels may lead to alterations in endometrial morphology, impair endometrial receptivity, and cause premature opening of the WOI. Such dysregulation can result in asynchrony between embryonic development and endometrial maturation, ultimately compromising pregnancy outcomes (58–61). In this study, P4 levels were significantly higher in COH mice than in the CTR group across all examined WOI time points, indicating a deviation from the physiological state observed in controls. EA intervention effectively reduced P4 levels, bringing them closer to the levels seen in the CTR group, a finding consistent with the report by Hu et al. (8). Based on these experimental data, we speculate that the surge in P4 likely stems from multiple simultaneous follicular secretions and enhanced luteal activity induced by COH. EA may partially restore hormonal homeostasis by strengthening regulation of the hypothalamic-pituitary-gonadal axis, thereby improving endometrial receptivity (62–64).

Gene microarray analysis has suggested that the beneficial effects of EA on endometrial receptivity in COH rats are closely associated with changes in the expression of circadian clock genes (65). This is in line with previous evidence. For example, re-analysis of human endometrial microarray datasets has revealed significant alterations in the expression of core clock genes CRY1/2 and PER1/2 during the WOI in patients with recurrent implantation failure, further highlighting the key role of clock genes in regulating endometrial receptivity (30). At the molecular level, mammalian circadian rhythms are driven by transcription-translation feedback loops formed by core clock genes (66). Essential components of this machinery include circadian locomotor output cycles kaput (CLOCK), brain and muscle ARNT-like 1 (BMAL1), PER1/2/3, and CRY1/2 (67). In addition, FBXL3 and CSNK1E are critical regulators of the core clock oscillator: FBXL3 mediates the ubiquitination and degradation of CRY proteins, thereby precisely controlling the circadian period (68, 69), whereas CSNK1E phosphorylates PER proteins to regulate rhythm length (70). Together, these genes and regulatory factors maintain the accuracy and stability of circadian rhythms. In our study, COH disrupted the circadian rhythmic expression of the clock genes *Per1*, *Cry1*, and *Cry2* and their regulators *Fbxl3* and *Csnk1e* along the HPOU axis during the WOI. EA intervention effectively restored the disordered rhythmic expression patterns of these genes.

Mammalian circadian rhythms are coordinated by both the central clock in the hypothalamic SCN and peripheral clocks. AVP neurons are densely distributed in the shell region of the SCN; their localization in this study, as confirmed by immunofluorescence, is consistent with the findings of Shan et al. (71). As key pacemaker components of the SCN network, AVP neurons regulate circadian oscillations by driving the rhythmic release of neuropeptides (72). Our data showed that EA exerted its most prominent effect on improving endometrial receptivity at WOI-ZT4 in COH mice. We therefore focused on WOI-ZT4 to further explore whether EA modulates peripheral clocks via AVP neurons and thereby influences endometrial receptivity.

The results showed that AVP neuropeptide expression in the SCN was reduced during the WOI−ZT4 period in COH mice, whereas EA enhanced its expression, suggesting that EA may activate AVP neurons in the SCN. Previous studies have confirmed that AVP neurons in the female rat SCN are closely associated with the expression of hypothalamic-pituitary-gonadal axis−related clock genes (73–75). Consistent with this, the present study found that EA improved the disrupted expression of HPOU−axis clock genes, reduced serum P4 levels and the P4/E2 ratio, and significantly enhanced endometrial receptivity at WOI−ZT4.

Previous evidence indicates that AVPR1A is an important AVP receptor for maintaining circadian rhythms (76, 77). In this study, the regulatory trend induced by the AVPR1A agonist resembled that of EA. It should be noted, however, that the agonist used is a modified peptide whose ability to cross the blood-brain barrier has not been verified; therefore, its effects may not be entirely attributable to direct action on AVP neurons in the SCN. Given the widespread expression of AVPR1A in peripheral reproductive tissues such as the ovary and uterus (78–80), the agonist may influence endometrial receptivity by modulating peripheral hormonal responses or the endometrial microenvironment. Future studies employing intracerebroventricular administration, tissue−specific knockout, or peripheral blockade strategies could help distinguish between central and peripheral mechanisms of AVPR1A−related effects.

**Figure 8:**
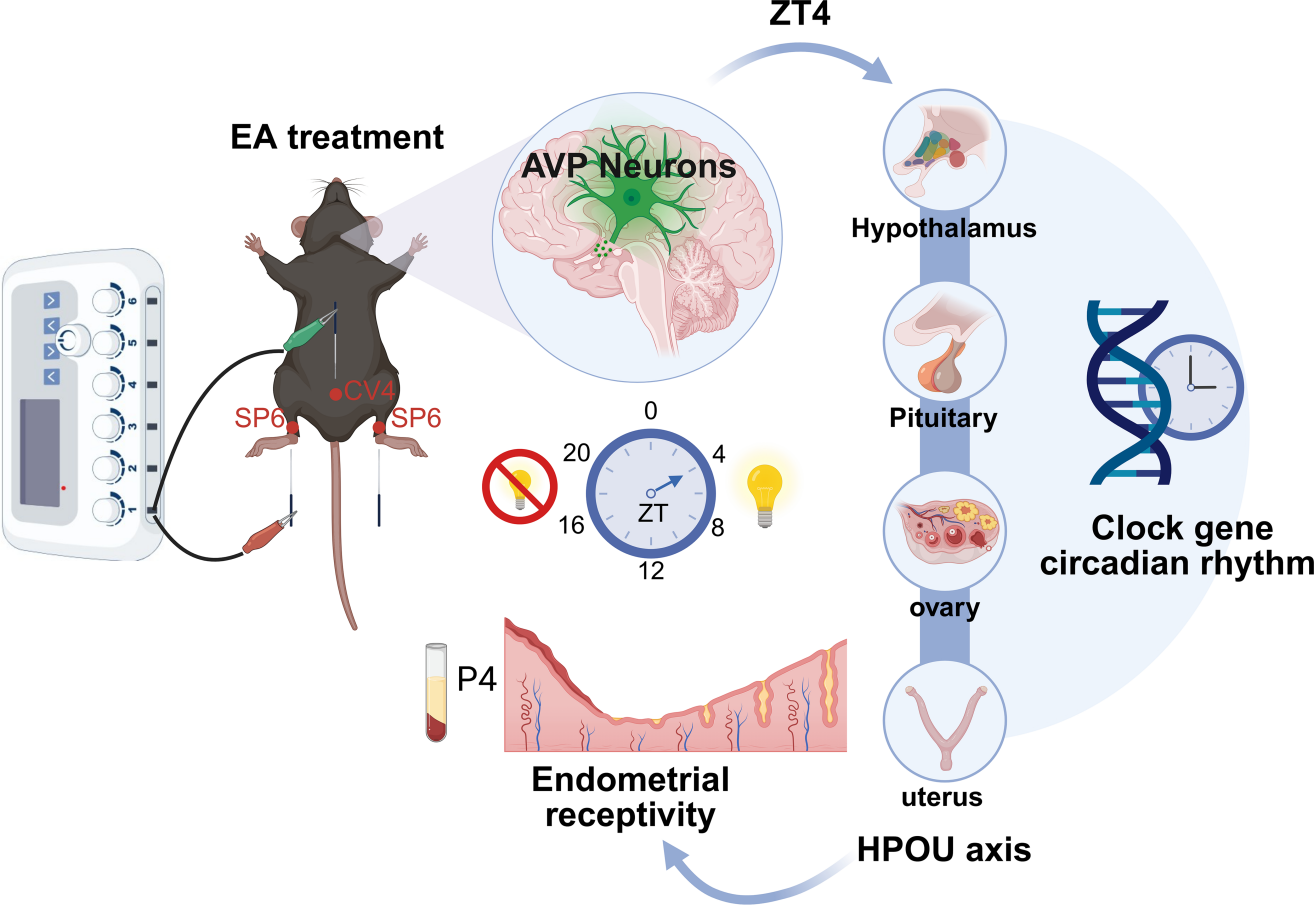
Graphical abstract. EA may improve endometrial receptivity at WOI-ZT4 in COH mice by activating AVP neurons in the central SCN clock, thereby regulating the expression of HPOU-axis clock genes in peripheral tissues and modulating P4 levels (created with https://biorender.com).

Furthermore, the AVPR1A antagonist attenuated, but did not completely reverse, EA−induced changes in some parameters, suggesting that the mechanism by which of EA improves endometrial receptivity does not rely exclusively on a single signaling pathway. As a holistic regulatory intervention, EA may exert its effects through multiple pathways, including modulation of the somatosensory−autonomic nervous system (81), improvement of endometrial structure (82), enhancement of endometrial blood flow and angiogenesis, and regulation of the immune−inflammatory microenvironment (83–85). In addition, AVP may indirectly influence HPOU axis function via other receptor subtypes or peripheral pathways, such as ovarian paracrine signaling and immune modulation (86, 87). In summary, the present study suggests that EA may improve endometrial receptivity during the WOI−ZT4 phase in COH mice by activating AVP neurons in the hypothalamic SCN and coordinately regulating the expression of peripheral HPOU−axis clock genes and the hormonal milieu (**Figure 8**). These findings provide new experimental evidence for understanding the circadian mechanisms underlying acupuncture−mediated regulation of reproductive function.

Despite these findings, this study has several limitations. First, the COH mouse model used here differs substantially from humans in reproductive system structure and physiology, which may limit the translational potential of our results. Future studies should prioritize validation of these mechanisms using human-derived endometrial organoids or non-human primate models. Second, the current assessment of clock gene circadian rhythms is based on independent sampling points from different individuals, which may introduce inter-individual variability and weaken data continuity. In future studies, longitudinal *in vivo* sampling of the same biological specimen could be used to obtain continuous data from the same sample (71, 88).

## 5 Conclusion

The results of this study indicate that EA improves endometrial receptivity in COH mice during the WOI-ZT4 period. This effect may be related to the activation of AVP neurons in the SCN and the restoration of expression of the peripheral tissue HPOU axis clock genes Per1, Cry1, and Cry2. EA also synergistically regulates serum P4 and P4/E2 levels, thereby jointly contributing to the improvement of endometrial receptivity under COH conditions.

## Data Availability

The raw data supporting the conclusions of this article will be made available by the authors, without undue reservation.
